# Alteration of hippocampal parvalbumin interneurons underlies memory impairment in rat model of Parkinson's disease

**DOI:** 10.3389/fnbeh.2025.1749815

**Published:** 2026-01-20

**Authors:** Ljiljana Radovanovic, Jasna Saponjic, Jelena Petrovic

**Affiliations:** Department of Neurobiology, Institute for Biological Research - Sinisa Stankovic, National Institute of Republic of Serbia, University of Belgrade, Belgrade, Serbia

**Keywords:** hippocampus, Parkinson's disease, parvalbumin-expressing neurons, rat, recognition memory, spatial memory

## Abstract

Cognitive decline is a major non-motor symptom in patients with Parkinson's disease (PD) that can be present as early as the prodromal stage. As a multisystem neurodegenerative syndrome, PD is associated with disturbances in various neurotransmitters, including dopamine, acetylcholine, serotonin, noradrenaline, glutamate, and gamma-aminobutyric acid (GABA). While the roles of dopaminergic and cholinergic deficiencies in cognitive impairment in PD are well documented, the contribution of the GABAergic system is less clear. We investigated spatial and recognition memory, along with changes in hippocampal GABAergic parvalbumin-positive (PV+) neurons, in distinct rat models of PD neuropathology. PD cholinopathy was induced by bilateral pedunculopontine tegmental nucleus (PPT) lesion, hemiparkinsonism was induced by unilateral substantia nigra pars compacta (SNpc) lesion, and hemiparkinsonism with PD cholinopathy was induced by unilateral SNpc and bilateral PPT lesions. Behavioral tests were conducted 14 and 42 days after lesions and included assessments of spatial memory (spatial habituation test), recognition memory (novel object recognition test), and measurements of motor activity (open field test). Motor function was preserved in all PD models. We observed delayed impairments in spatial and recognition memory in PD cholinopathy, and persistent impairment in spatial memory in hemiparkinsonism, although hippocampal PV expression remained unchanged over time. In hemiparkinsonism with PD cholinopathy, persistent spatial memory impairment was followed by delayed recognition memory deficits, along with hippocampal PV suppression, which was functionally linked to recognition memory impairment. Our results show that different PD neuropathologies underlie different memory impairments in rats. While dopaminergic denervation plays an important role in impairing spatial memory from the prodromal stage of PD, cholinergic denervation impairs recognition memory in a delayed manner. However, only their synergistic dysfunction alters hippocampal GABAergic PV+ neuron-mediated inhibitory transmission during PD progression, which was correlated with memory impairment.

## 1 Introduction

Parkinson's disease (PD), which affects about 1% of the elderly population (Ou et al., 2021), is the second most common neurodegenerative disorder, predominantly characterized by a range of motor symptoms (Aarsland et al., 2021). Over the past decades, PD research has focused on the selective loss of dopaminergic (DA) neurons in the *substantia nigra pars compacta* (SNpc) and the consequent striatal DA deficiency underlying these motor symptoms (Gonzalez-Latapi et al., 2021). However, PD is more than a dopaminergic disease with motor impairments. Multiple mechanisms and pathway dysfunctions contribute to the pathogenesis of PD. It has been suggested that, in addition to dopaminergic system, many other brain systems also undergo pathological alterations that contribute to the non-motor symptoms and overall PD pathology (Postuma and Berg, 2019; Schapira et al., 2017). Therefore, PD is now recognized as a heterogeneous, multisystem neurodegenerative disorder comprising a wide range of both motor and non-motor symptoms.

Although clinical diagnosis of PD still depends on the presence of motor deficits, it has been suggested that these diagnostic features may be preceded by non-motor symptoms (Postuma and Berg, 2019; Schapira et al., 2017). In recent years, scientific interest has shifted from motor to non-motor manifestations, which appear to be preclinical features and a major component of prodromal PD, linked to the initial (pre-motor) stage of the disease. It is now known that the neurodegenerative processes underlying PD start long before the onset of motor deficits (Postuma and Berg, 2019). Symptoms such as impaired olfaction, gastrointestinal abnormalities, sleep disturbances, visual alterations, and cognitive and mood disorders may be present several years before the emergence of motor deficits. These non-motor symptoms are frequent and become increasingly prevalent as the disease progresses (Gonzalez-Latapi et al., 2021).

Cognitive impairment is a characteristic non-motor feature of PD that can be present as early as the prodromal stage of the disease. Up to six times more common in PD patients than in age-matched controls (Das et al., 2019), cognitive deficit is recognized as an integral part of PD that contributes significantly to the overall disease burden. As one of the most disabling non-motor symptoms, cognitive decline is associated with reduced quality of life and distress for both patients with PD and their caregivers (Aarsland et al., 2021). Similar prodromal cognitive changes, affecting hippocampal memory performance, have been documented in patients with Alzheimer's disease, and deficit in dopaminergic signaling, particularly midbrain dopaminergic depletion, has been suggested as a common feature with PD (Krashia et al., 2019; Manca et al., 2025).

Cognitive deficits in PD show high variability in severity, rate of progression, and types of cognitive domains affected (Aarsland et al., 2021). The cognitive profile in PD can range from normal cognition to mild cognitive impairment (MCI) and dementia. Early cognitive changes, such as subjective cognitive decline (SCD), are usually subtle and asymptomatic. As the disease progresses, these disorders become more severe, leading to MCI. MCI is associated with impairments in several cognitive domains including executive function, attention, visuospatial abilities and memory (Aarsland et al., 2021; Jellinger, 2024), and refers to a state of gradual cognitive decline confirmed by neurophysiological testing, but not yet severe enough to affect activities of daily living (Das et al., 2019). As memory deficits continue to progress, most patients will eventually be diagnosed with dementia (PDD), a cognitive impairment that affects at least two of four cognitive domains, and is severe enough to disrupt daily functioning (Gonzalez-Latapi et al., 2021; Jellinger, 2024).

As a pre-dementia stage, MCI is present in about 25% of patients at the time of PD diagnosis, and more than 80% of patients will progress to dementia over 20 years (Aarsland et al., 2021). Although cognitive deficits can occur in multiple domains, the most common cognitive symptom reported by early diagnosed PD patients and confirmed by neuropsychological assessments is episodic (recognition) memory impairment, particularly in immediate and delayed free recall (Das et al., 2019; Muslimovic et al., 2005). According to experimental psychologists, recognition memory consists of two subcomponents: recollection (referred to as remembering) and familiarity (referred to as knowing) which are considered to depend primarily on the hippocampus and perirhinal cortex, respectively (Das et al., 2019; Jacoby and Dallas, 1981; Yonelinas, 2001). Neurodegenerative processes underlying cognitive impairment in PD affect multiple brain regions and lead to dysfunction in many neuronal networks involved in cognitive, attentional, memory and behavioral functions (Calabresi et al., 2013; Costa et al., 2012; Jellinger, 2023; Pendolino et al., 2014). There is evidence that brain regions implicated in recognition memory are subject to atrophy in PD patients. Several studies have demonstrated a strong association between PD-related MCI and hippocampal atrophy (Camicioli et al., 2003; Mak et al., 2015; Segura et al., 2014) and have suggested that disrupted recollection, but not familiarity, underlies episodic memory deficit in PD patients with respect to healthy controls (Algarabel et al., 2010; Rodríguez et al., 2014).

Despite intensive research over the past two decades, the exact mechanisms underlying cognitive decline in PD remain largely unclear. Cognitive decline results from structural or functional abnormalities in the cortex, and one mechanism that may explain cognitive impairment in PD is neurotransmitter dysregulation (Kehagia et al., 2010; Ray and Strafella, 2012). Current findings suggest that PD is a multisystem neurodegenerative syndrome related to disturbances in various neurotransmitters such as dopamine (DA), acetylcholine, serotonin, noradrenaline, glutamate and gama-aminobutyric acid (GABA) (Murueta-Goyena et al., 2019; Sanjari Moghaddam et al., 2017). While the roles of dopaminergic and cholinergic deficiencies in cognitive impairment in PD are well documented (Chen and Zhang, 2024; McKinley et al., 2019), the contribution of the GABAergic system is less clear.

GABA is the main inhibitory neurotransmitter in the brain, primarily released by local interneurons to regulate cortical and subcortical microcircuits. GABA signaling controls a wide range of physiological functions including sensory processing, cognition, information processing and sleep (Błaszczyk, 2016; Murueta-Goyena et al., 2019; Sohal et al., 2009). GABAergic neurons are directly involved in memory and learning (Magrinelli et al., 2016; Marín, 2012; Verret et al., 2012). Moreover, regulation of cognitive function depends heavily on the interaction between excitatory neurotransmitters and GABA inhibition, with parvalbumin-expressing (PV+) neurons playing a central role in maintaining this excitatory/inhibitory (E/I) balance (Smeralda et al., 2024). PV+ neurons are a major type of GABAergic inhibitory interneuron in the brain, characterized by short action potential duration and the ability to fire at high frequencies (Hijazi et al., 2020). These neurons are densely connected to other PV+ and non-PV+ neurons through electrical and chemical synapses, contributing to integrated inhibitory control of both local circuits and remote neuronal networks (Hijazi et al., 2020). GABAergic PV+ neurons are crucial for memory processes, particularly in spatial memory consolidation in the hippocampus (Ognjanovski et al., 2017; Verret et al., 2012; Yi et al., 2014).

GABAergic neurons may be severely affected in various neurodegenerative diseases (Alharbi et al., 2024). Alterations in the GABAergic system have been observed in PD patients and are associated with the development of cognitive dysfunction. Postmortem studies have shown decreased mRNA levels of GAD67 and PV in the prefrontal cortex of PD patients, indicating reduced neuronal synthesis and release of GABA, and thus a reduction of inhibition in the cortex (Lanoue et al., 2010). In addition, evidence suggests that alteration of inhibitory transmission in the hippocampus, particularly by GABAergic PV+ neurons, is linked to spatial memory deficits. Early treatment of GABAergic PV+ neuron hyperactivity may be clinically relevant for preventing memory decline, local network hyperexcitability, and delaying the progression of neurodegenerative diseases such as Alzheimer's disease (Hijazi et al., 2020).

Considering the above findings, which indicate the high importance of the GABAergic system in the pathophysiology of PD, we hypothesize that dysregulation of GABA signaling through alteration of PV+ neurons could be the underlying mechanism for memory deficits in PD. Therefore, in this study, we investigated spatial and recognition memory, along with the changes in hippocampal GABAergic PV+ neurons in distinct experimental models of PD neuropathology (rat model of PD cholinopathy, hemiparkinsonism and hemiparkinsonism with PD cholinopathy).

## 2 Materials and methods

Experiments were conducted on 53 adult male Wistar rats (each two and a half months old, weighing 250–290 g), randomly assigned to four experimental groups: control (*n* = 17), PD cholinopathy (bilateral *pedunculopontine tegmental nucleus* (PPT) lesion, *n* = 12), hemiparkinsonism (unilateral SNpc lesion, *n* = 11), and hemiparkinsonism with PD cholinopathy (unilateral SNpc/bilateral PPT lesion, *n* = 13).

**Figure 1:**
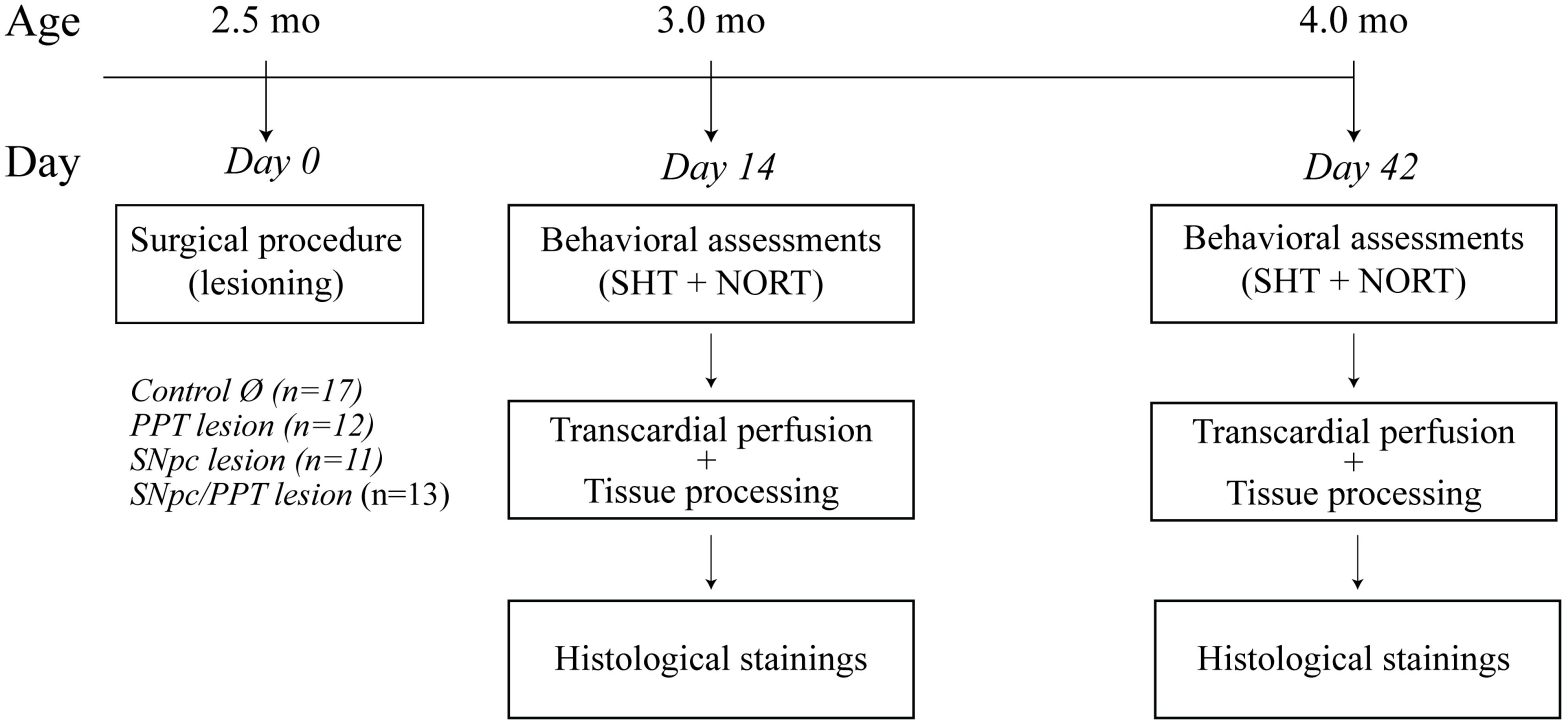
Experimental timeline. SHT, Spatial habituation test; NORT, Novel object recognition test.

We investigated spatial and recognition memory, as well as basal locomotor activity, in distinct rat models of PD neuropathology, along with alteration in PV+ expression within the hippocampus 14 and 42 days after lesions. Two post-lesion time points were selected to track lesion-induced neuropathology over time. Day 14 corresponds to the onset of neuropathology, and all alterations observed at day 14 were considered prodromal. Day 42 corresponds to the progression of neuropathology, and all alterations observed at day 42 were considered delayed. The experimental timeline is shown in Figure 1.

After surgery and throughout the experimental protocol, animals were housed individually in custom-made clear Plexiglas cages (30 × 30 × 30 cm) and maintained on a 12-hour light–dark cycle (lights on at 7 a.m.; lights off at 7 p.m.) at 25 °C, with free access to food and water.

All the procedures were performed in accordance with the recommendations of EEC Directive (2010/63/EU) on the Protection of Animals Used for Experimental and Other Scientific Purposes, and the protocol was approved by the Ethical Committee for the Use of Laboratory Animals of the Institute for Biological Research “Sinisa Stankovic”—National Institute of Republic of Serbia, University of Belgrade (Approval No. 01-1926; 18/11/2021) and by the Veterinary Directorate, Department of Animal Welfare, Ministry of Agriculture, Forestry and Water Management of Republic of Serbia (Approval No. 323-07-10509/2020-05/1; 13/10/2020).

### 2.1 Surgical procedure for lesioning

Rats were anesthetized with ketamine/diazepam (50 mg/kg, i.p., Zoletil 50, Virbac France), and placed in a stereotaxic frame. All lesions were performed by stereotaxically guided microinfusions using a Digital Lab Standard Stereotaxic Instrument (Stoelting Co., Dublin, Ireland) with a Quintessential Stereotaxic Injector (Stoelting Co., Wood Dale, IL, USA) and a Hamilton syringe (1μL).

PD cholinopathy was induced by bilateral PPT lesion using ibotenic acid (IBO, Sigma-Aldrich, St. Louis, MO, USA). We infused 100 nL of 0.1 M IBO/0.1 M PBS bilaterally into the PPT (A/P: −7.8 mm from bregma; R/L: 1.9 mm from the sagittal suture; D/V: 7.0 mm from the brain surface, following Paxinos and Watson, 2005) as a continuous infusion over 60 s (Petrovic et al., 2013, 2021; Radovanovic et al., 2021).

Hemiparkinsonism was induced by a unilateral SNpc lesion using 6-hydroxy dopamine hydrobromide salt (6-OHDA, Sigma-Aldrich, St. Louis, MO, USA). We infused 1 μL of 6 μg/μL 6-OHDA, dissolved in ice-cold sterile saline (0.9% NaCl) and supplemented with 0.2% ascorbic acid as an anti-oxidant, into the right SNpc (A/P: −5.3 mm from bregma; R: 2.4 mm from the sagittal suture; D/V: 7.4 mm from the brain surface, following Paxinos and Watson, 2005). The 6-OHDA microinfusions was administered as a continuous infusion at 200 nL/min, over 5 min (Ciric et al., 2019; Petrovic et al., 2021; Radovanovic et al., 2021). To minimize uptake of 6-OHDA by noradrenergic neurons each rat received a bolus of desipramine hydrochloride (28.42 mg/kg, i.p., Sigma-Aldrich, Taufkirchen, Germany; pH 7.4) 30 min prior to microinfusion.

To induce hemiparkinsonism with PD cholinopathy, we performed double lesioning: a unilateral SNpc lesion and a bilateral PPT lesion (Ciric et al., 2019; Petrovic et al., 2021; Radovanovic et al., 2021).

After each microinfusion, the needle remained in the local brain tissue for 5 min to allow the solution to diffuse within the PPT or SNpc. For the bilateral PPT lesions, the Hamilton syringe needle was always washed after the first IBO microinfusion before infusing the contralateral PPT.

At the end of the surgical procedure, the scalp wounds were sutured and the rats were allowed to recover for 2 weeks. In contrast to above-described models of PD neuropathology, control rats were not subjected to any surgical procedure (intact control).

### 2.2 Behavioral assessments

**Figure 2:**
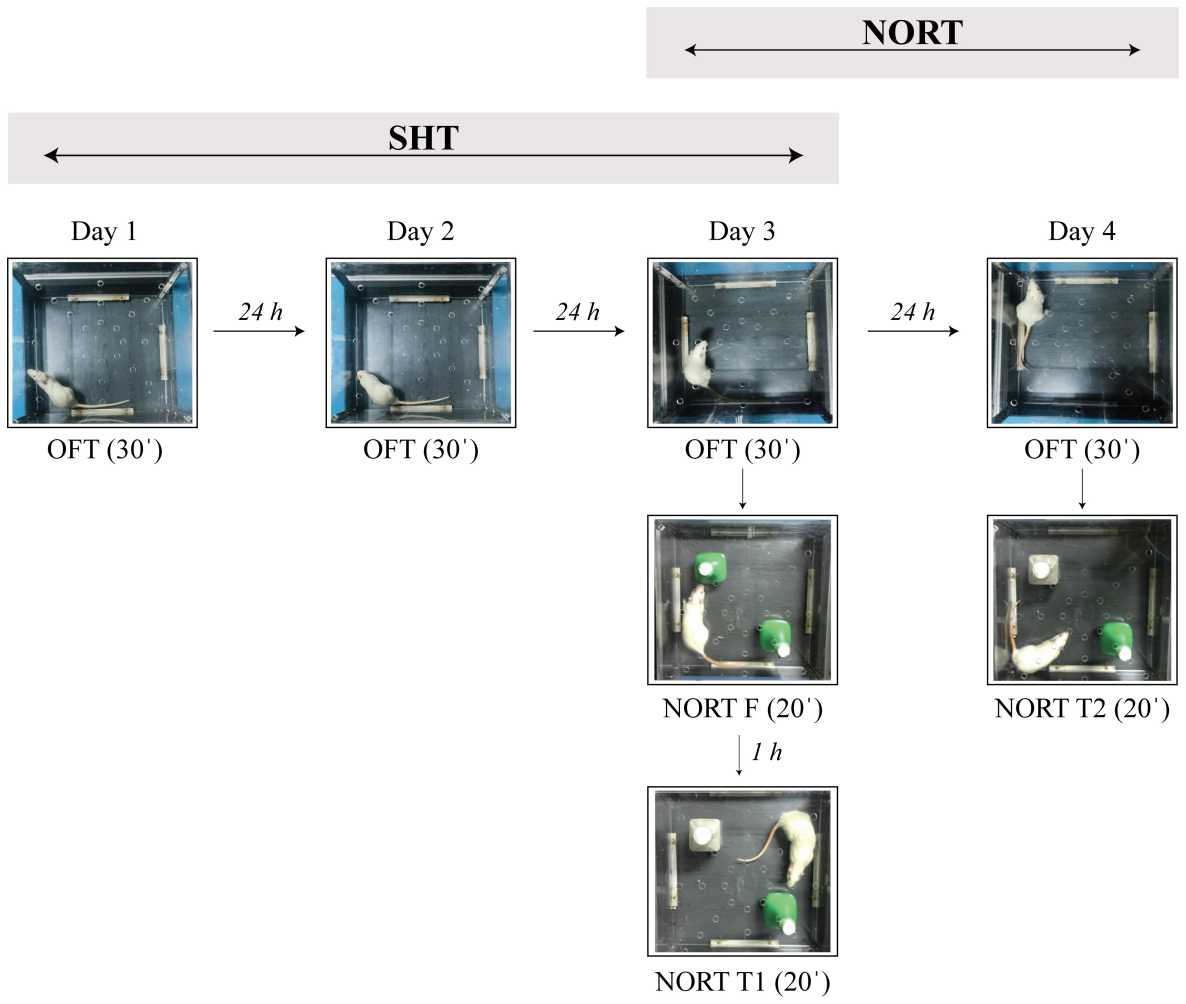
Experimental protocol for behavioral testing. The spatial habituation test (SHT) was conducted over three consecutive days (Day 1–Day 3; 30 min per test phase). The novel object recognition test (NORT) was performed after the SHT, which served as long habituation (Day 3–Day 4; 20 min per test phase). OFT, open field test; NORT F, familiarization phase; NORT T1, test phase 1 (short-term memory test); NORT T2, test phase 2 (long-term memory test).

The behavioral experiments were conducted during the rats' inactive circadian phase (from 9 a.m. to 3 p.m.), 14 and 42 days after the lesions. Behavioral tests included assessments of spatial and non-spatial memory, as well as measurements of motor activity. We used two non-aversive memory tasks: the spatial habituation task (SHT) and the novel object recognition task (NORT). Both SHT and NORT are hippocampus-dependent tasks based on rodents' natural preference for novelty (Leussis and Bolivar, 2006; Ennaceur, 2010; Cohen and Stackman, 2015). All behavioral tests were conducted in Opto-Varimex cages (44.2 × 43.2 × 20 cm; Columbus Instruments, OH), with no spatial cues present. Between each subject, fecal boli and urine were removed, and all surfaces (arenas and objects) were wiped with 20% ethanol, followed by distilled water, and allowed to dry before the next test session. The protocol for behavioral testing is shown in Figure 2.

#### 2.2.1 Spatial habituation task

The SHT was conducted over three consecutive sessions, each separated by 24-h interval (inter-session habituation; Figure 2. Day 1–Day 3), and served as an indirect measure of spatial memory abilities. Before each test, the animals in their home cages were allowed to habituate to the experimental room for 30 min. During each session, the rats were exposed to the open arenas (following the principle of an open field test—OFT) and monitored for 30 min using the Opto-Varimex system with Auto-Track software (Columbus Instruments, Columbus, OH, USA). Basal locomotor activity was assessed based on the distance traveled in the open arenas during habituation over 30 min and in 5-min intervals. Habituation was evaluated based on the inter-session scores of basal locomotor activity (Ciric et al., 2018, 2019; Petrovic et al., 2021; Radovanovic et al., 2021).

#### 2.2.2 Novel object recognition task

Since we previously observed impaired spatial memory abilities in the hemiparkinsonian rats (Ciric et al., 2019; Petrovic et al., 2021) we decided to use a modified NORT protocol with extended habituation (30 min per day over 3 days) to reduce the influence of spatial (arena) context. Additionally, the modifications included a prolonged sample phase (20 min) to ensure stronger, more deeply encoded object memory that is hippocampus-dependent (Cohen and Stackman, 2015; Pavković et al., 2018). Therefore, NORT was performed following the SHT (Figure 2, Day 3–Day 4) and consisted of three phases (20 min each): the familiarization phase (NORT F, Day 3), test phase 1 (NORT T1, Day 3, short-term memory test, retention interval of 60 min) and test phase 2 (NORT T2, Day 4, long-term memory test, retention interval of 24 h). Following the familiarization phase, animal behavior during NORT T1 reflected recognition and exploration of novelty (new learning during retrieval), while during NORT T2 it reflected memory of previously experienced novelty. NORT T2 was conducted using the same objects in the same positions as during NORT T1. Before each test, the animals in their home cages were allowed to habituate to the experimental room for 30 min.

Animal behavior during the NORT was recorded by video camera and subsequently evaluated quantitatively by an observer blinded to treatment. Exploration activity was assessed as the time spent exploring each object during 20-min sessions and in 5-min intervals. Based on these data, we determined for each test phase and experimental group: (1) general exploration activity during NORT (% of animals within the corresponding experimental group actively exploring the objects during each 5-min interval) and (2) the timeline of explorative activity (% of time spent in object exploration during each 5-min interval relative to the total exploration time during the 20-min session, taken as 100%). Recognition memory was measured by the preference index (PI) (Antunes and Biala, 2012; Gulinello et al., 2019). The PI was calculated as PI = T_n_/(T_f_ + T_n_), where T_n_ is the time with the novel object and T_f_ is the time with the familiar object. The resulting PI value ranges from 0 (preference for the familiar object) to 1 (preference for the novel object), with values around 0.5 indicating no preference for either object (Hammond et al., 2004; Antunes and Biala, 2012).

Although the PI was initially calculated for each 5-min interval and the total 20-min period, the final analysis included only the PI for the first 5-min interval. Our subsequent analysis of explorative activity showed that the most intensive exploration occurred during the first 5-min interval, regardless of experimental group or test phase. During this period of time all animals were active and explored the objects. From this period to the end of the sessions, we observed a global decrease in explorative activity, with fewer animals exploring and less time spent in exploration. Therefore, hippocampus-dependent short- and long-term recognition memory was measured specifically during the first 5-min interval.

### 2.3 Tissue processing for histology

At the end of each behavioral assessment (14 and 42 days after lesions) the rats were sacrificed for further histology (lesion identification and PV immunohistochemistry). All animals were deeply anesthetized with ketamine/diazepam (50 mg/kg i.p. Zoletil 50, Virbac France) and perfused transcardially with 0.9% saline, followed by 4% paraformaldehyde (PFA, Sigma Aldrich, Taufkirchen, Germany) in 0.1 M PBS (pH 7.4) and finally with 10% sucrose solution in 0.1 M PBS. The brains were removed and immersed in 4% PFA overnight, then placed in a 30% sucrose solution for several days. The brains were serially sectioned on a cryostat (Leica, Wetzlar, Germany) into coronal 40 μm-thick sections, and the free-floating sections were stored in a cryoprotective buffer for further use (Ciric et al., 2019; Petrovic et al., 2021; Radovanovic et al., 2021).

### 2.4 Lesion identification and quantification

The PPT lesion was identified using NADPH–diaphorase histochemistry (Paxinos et al., 2009). As previously described (Ciric et al., 2018; Petrovic et al., 2013, 2021) the free-floating sections were rinsed in 0.1 M PBS (pH 7.4) and incubated for 1 h at 37°C in a staining solution containing NADPH reduced tetrasodium salt (Serva, Heidelberg, Germany) and dimethyl sulfoxide (DMSO, Sigma-Aldrich, Taufkirchen, Germany) dissolved in substrate solution. The substrate solution contained nitro blue tetrazolium chloride (NBT, Serva, Heidelberg, Germany) and 5-bromo-4-chloro-3-indolyl phosphate (BCIP, Serva, Heidelberg, Germany) dissolved in substrate buffer at pH 9.5 (0.1 M Tris, 100 mM NaCl, 5 mM MgCl_2_). Background staining induced by endogenous alkaline phosphatase was reduced by 2 mM levamisole (Sigma-Aldrich, Taufkirchen, Germany). Finally, all sections were mounted on slides, placed in a clearing agent (Xylene, Zorka Pharma, RS), coverslipped using DPX (Sigma Aldrich, Burlington, MA, USA), and examined under a Zeiss Axiovert microscope with a camera (Zeiss, Jena, Germany).

The SNpc lesion was identified by tyrosine hydroxylase (TH) immunohistochemistry (Ciric et al., 2019; Petrovic et al., 2021). Brain sections were thoroughly rinsed with 0.1 M PBS. Endogenous peroxidase activity was neutralized using 3% hydrogen peroxide/10% methanol/0.1 M PBS for 15 min. Non-specific binding was prevented by incubating the sections for 60 min in 5% normal donkey serum (D9663, Sigma-Aldrich, Burlington, MA, USA) in 0.1 M PBS at room temperature (Ciric et al., 2019; Petrovic et al., 2021). The sections were then incubated for 48 h at +4°C with a primary mouse monoclonal anti-TH antibody (dilution 1:16000, T2928, Sigma-Aldrich, Burlington, MA, USA) in PBS with 0.5% Triton X-100 (Sigma-Aldrich, Burlington, MA, USA), followed by 90 min in polyclonal rabbit anti-mouse immunoglobulin (dilution 1:100, P0260, Agilent Dako, Glostrup, Denmark). Between each immunolabeling step, the sections were washed in fresh 0.1 M PBS (3 x 5 min). Immunoreactive signals were visualized using a diaminobenzidine (DAB) solution (1% 3,3′-diaminobenzidine (11208, Acros organics, Geel, Belgium)/0.3% hydrogen peroxide/0.1 M PBS). All sections were mounted on slides, dehydrated through increasing concentrations of ethanol (70%, 96%, 100%, Zorka Pharma, Sabac, RS), cleared in xylene (Xylene, Zorka Pharma, Sabac, RS), coverslipped with DPX (Sigma-Aldrich, Burlington, USA), and examined under a Leica light microscope with a camera (Leica, Wetzlar Germany). To test the specificity of the immunolabeling, the primary antibody was omitted in control experiments.

Cholinergic and/or dopaminergic neuronal loss was quantified by counting the NADPH–diaphorase or TH positively stained cells using ImageJ 1.46 software (Ciric et al., 2018, 2019; Petrovic et al., 2013, 2021). Tissue samples included SNpc sections within the stereotaxic range of 5.20–5.70 mm posterior from bregma and PPT sections within the stereotaxic range of 7.50–8.00 mm posterior from bregma (Paxinos et al., 2009). The neuronal losses were expressed with respect to the mean control absolute number, which was taken as 100%. The unilateral SNpc lesions were quantified with respect to its corresponding contralateral SNpc. The bilateral PPT lesions (each brain side) were quantified with respect to controls (Ciric et al., 2018, 2019; Petrovic et al., 2013, 2021).

### 2.5 Immunohistochemistry for PV

The free-floating hippocampal brain sections were thoroughly rinsed with 0.1 M PBS (pH 7.4). Non-specific binding was prevented by incubating the sections in 3% hydrogen peroxide/10% methanol/0.1 M PBS for 15 min, followed by 5% normal donkey serum/0.1 M PBS for 60 min at room temperature. The sections were then incubated overnight at +4 °C with mouse monoclonal anti-PV antibody (dilution 1:2000, P3088, Sigma-Aldrich, Burlington, MA, USA) (Radovanovic et al., 2021, 2023; Rajkovic et al., 2019). The primary antibody was diluted in PBS containing 0.5% Triton X-100. After three 5-min washes in 0.1 M PBS, the sections were incubated for 90 min with polyclonal rabbit anti-mouse immunoglobulin (dilution 1:100). Immunoreactive signals were visualized using a DAB solution. All sections were mounted on slides, dehydrated through increasing concentrations of ethanol (70%, 96%, and 100%), placed in a clearing agent, coverslipped with DPX, and examined under a Leica light microscope with a camera. To test the specificity of immunostaining, the primary antibody was omitted in control experiments.

#### 2.5.1 Quantification of PV immunostaining

PV immunoreactivity within the hippocampal dentate gyrus (DG) was quantified using ImageJ 1.46 software (NIH, Bethesda, MD, USA,) by counting the number of PV immunoreactive (PV+) neurons (Radovanovic et al., 2021, 2023; Rajkovic et al., 2019). Tissue samples included hippocampal sections within the stereotaxic range of 3.10–4.60 mm posterior from bregma (Paxinos et al., 2009). For all experimental groups, the number of PV+ neurons was counted for each brain side and pooled for each experimental group.

### 2.6 Statistical analyses

All data in the text and in the figures are presented as mean ± standard error of the mean (SEM) with column bars and were statistically analyzed using IBM SPSS Statistics for Windows, Version 25.0 (IBM Corp., Released 2017, Armonk, NY). Cholinergic deficit between PD cholinopathy and hemiparkinsonism with PD cholinopathy, as well as dopaminergic deficit between hemiparkinsonism and hemiparkinsonism with PD cholinopathy, were analyzed using the nonparametric Mann-Whitney U test for pairwise comparisons. For basal locomotor activity, detailed statistical anlysis was performed for the data obtained during the first 5 min of testing (the period of most intensive exploration of a new environment) and for the total scores, using nonparametric single factor Kruskal–Wallis ANOVA with the Mann–Whitney U post hoc test. SHT data were analyzed by nonparametric single factor Kruskal–Wallis ANOVA with the Mann–Whitney U post hoc test. General exploration activity was assessed based on a threshold that was set at 50%. When group activity during given 5-min interval was at or above 50% (≥50% of animals within the group were active), it was considered active. When the group activity was below 50%, it was considered not active. Time-line of explorative activity was analyzed by nonparametric single factor Kruskal–Wallis ANOVA with the Mann–Whitney U post hoc test. To analyze preference during NORT, PIs were compared to chance levels (PI = 0.5) to confirm the preference using a one-sample *t*-test. Normality of the NORT PIs data set was assessed by the Shapiro-Wilk test. PV quantification data were analyzed by nonparametric one-way Kruskal–Wallis ANOVA with the Mann–Whitney U post hoc test. Pearson's correlation coefficient was used for correlation analysis. For all statistical analyses, the accepted level of significance was *p* ≤ 0.05.

## 3 Results

### 3.1 Lesion identification and quantification

**Figure 3:**
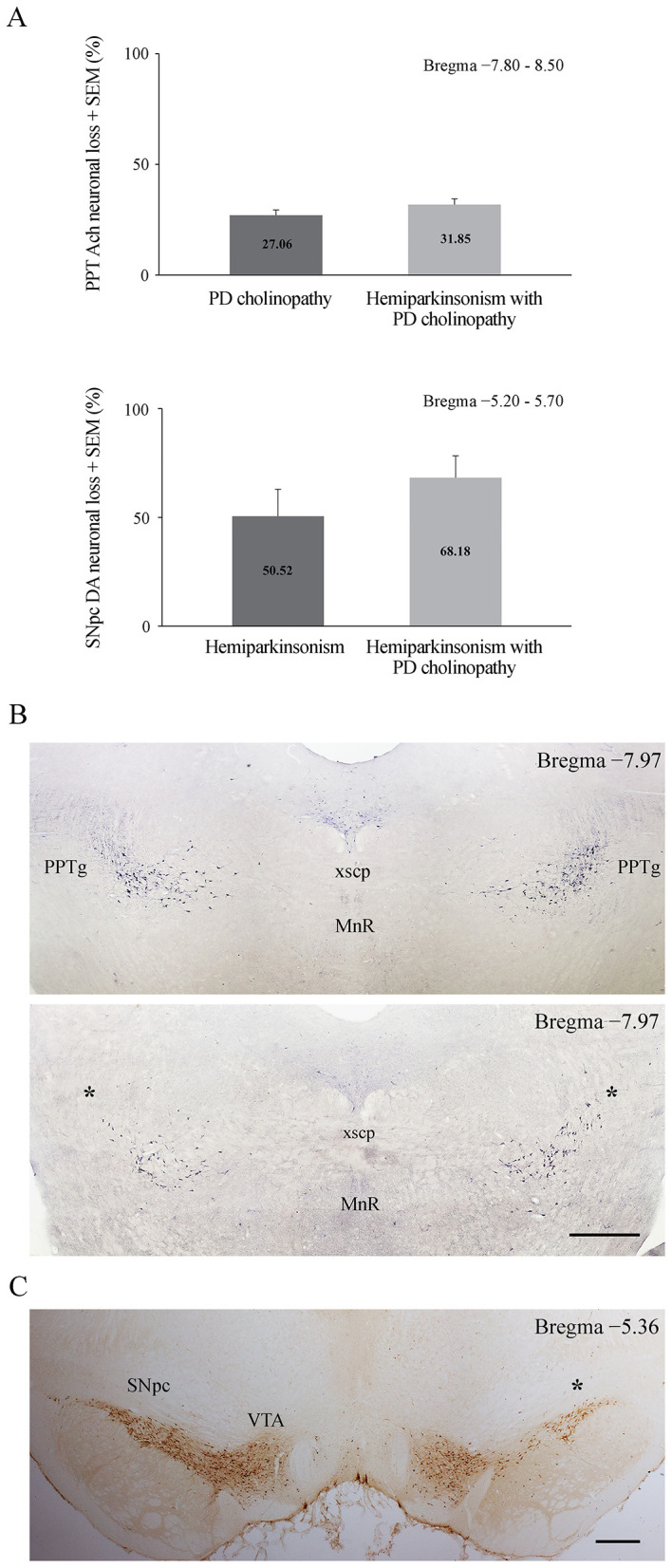
Histological identification and quantification of lesions. **(A)** Quantification of cholinergic and dopaminergic neuronal loss in PD cholinopathy (PPT lesion, *n* = 12), hemiparkinsonism (SNpc lesion, *n* = 8) and hemiparkinsonism with PD cholinopathy (SNpc/PPT lesion, *n* = 12). There was no difference in cholinergic deficit between PD cholinopathy and hemiparkinsonism with PD cholinopathy (z = −1.31; *p* = 0.19) and no difference in dopaminergic deficit between hemiparkinsonism and and hemiparkinsonism with PD cholinopathy (z = −1.14; *p* = 0.25); **(B)** Individual examples of histological identification of cholinergic deficit (identified by NADPH-diaphorase staining) in a model of hemiparkinsonism with PD cholinopathy, Partial bilateral lesion of cholinergic neurons within the PPT (lower panel) compared to the intact (control) PPT (upper panel). PPTg, *pedunculopontine tegmental nucleus pars compacta*; xcsp, *decussation of the superior cerebellar peduncle*; MnR, *median raphe nucleus*; asterisks indicate the lesioned PPT. Scale bar: 200 μm; **(C)** Individual examples of histological identification of dopaminergic deficit (identified by TH immunochemistry) in a model of hemiparkinsonism with PD cholinopathy – Partial unilateral lesion of dopaminergic neurons within the right SNpc compared to the intact left SNpc. SNpc, *substantia nigra pars compacta;* VTA, *ventral tegmental area*; asterisk indicates the lesioned SNpc. Scale bar: 400 μm.

Before analyzing the behavioral data, we verified our experimental PD models by identifying the PPT and SNpc lesions. Histological identification and quantification of cholinergic and dopaminergic neuronal loss is shown in Figure 3. In the PPT lesioned rats the mean cholinergic neuronal loss was 27.06 ± 2.38% per each brain side (Figure 3A, PD cholinopathy). In the SNpc lesioned rats the mean dopaminergic neuronal loss was 50.52 ± 12.40 % (Figure 3A, hemiparkinsonism). In the SNpc/PPT lesioned rats the mean cholinergic neuronal loss was 31.85 ± 2.66 % per each brain side, whereas the mean dopaminergic neuronal loss was 68.18 ± 10.11 % (Figure 3A, hemiparkinsonism with PD cholinopathy). There was no statistically significant difference in the cholinergic deficit between PD cholinopathy and hemiparkinsonism with PD cholinopathy (z = −1.31; *p* = 0.19). In addition, there was no statistically significant difference in dopaminergic deficit between hemiparkinsonism and hemiparkinsonism with PD cholinopathy (z = −1.14; *p* = 0.25). As depicted by the individual examples of bilateral PPT lesion (Figure 3B, lower panel) and unilateral SNpc (Figure 3C), the lesions were incomplete but selective and always within the boundaries of the targeted nucleus. Any rat lacking a lesion was excluded from further analyses.

### 3.2 Basal locomotor activity in different rat models of PD

**Figure 4:**
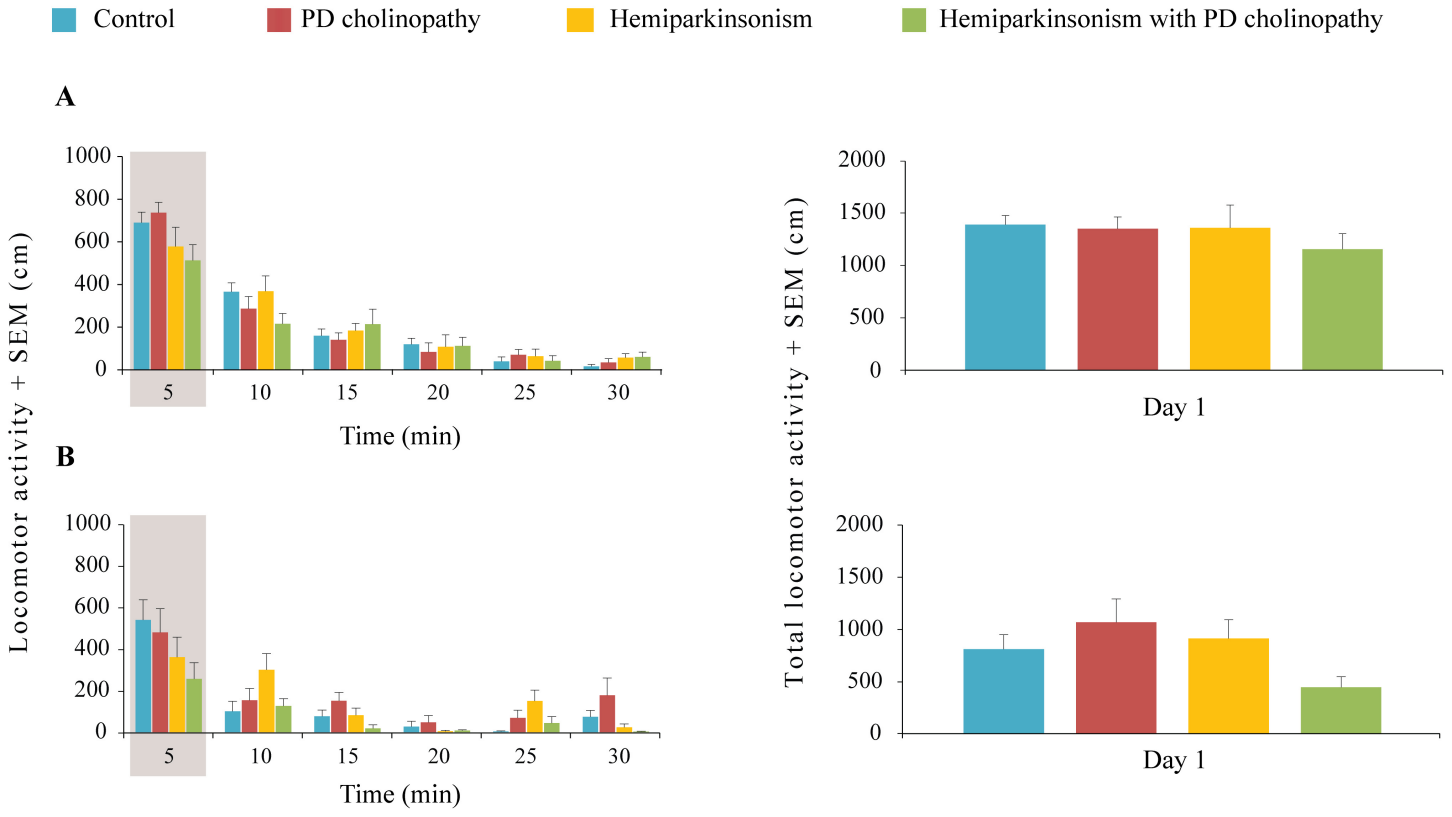
Basal locomotor activity in different rat models of PD neuropathology. **(A)** Spontaneous locomotor activity 14 days after lesions: time-dependent profiles for 5-min periods during the session (left panel) and total distance traveled during the overall 30-min session (right panel) in control (*n* = 14), PD cholinopathy (*n* = 10), hemiparkinsonism (*n* = 7) and hemiparkinsonism with PD cholinopathy (*n* = 6). Detailed statistical anlysis was performed for the data obtained during the first 5 min of testing (left pannel) and for the total scores (right pannel). There was no difference in locomotor activity between groups during the first 5-min interval (χ^2^ = 5.87; *p* = 0.12) and during overall 30-min session (χ^2^ = 2.01; *p* = 0.57); **(B)** Spontaneous locomotor activity 42 days after lesions: time-dependent profiles for 5-min periods during the session (left panel) and total distance traveled during the overall 30-min session (right panel) in control (*n* = 6), PD cholinopathy (*n* = 5), hemiparkinsonism (*n* = 5) and hemiparkinsonism with PD cholinopathy (*n* = 5). There was no difference in locomotor activity between groups during the first 5-min interval (χ^2^ = 4.93; *p* = 0.18) and during overall 30-min session (χ^2^ = 7.33; *p* = 0.07). There were no differences in locomotor activity between the groups (no motor deficit) at 14 and 42 days after lesions.

We examined basal locomotor activity in all experimental models of PD to identify any baseline differences in motor performance. We evidenced a global decline in locomotor activity during 30 min of the OFT in all experimental groups, indicating intra-session habituation, but without motor deficits (Figure 4). Specifically, there were no differences in locomotor activity among the experimental groups 14 and 42 days following lesions, for both the 5-min timelines (Figure 4A, χ^2^ ≥ 2.155; *p* ≥ 0.09;) and total locomotor activity (Figure 4B, χ^2^ ≥ 2.01; *p* ≥ 0.07).

### 3.3 Spatial memory impairment in different rat models of PD

**Figure 5:**
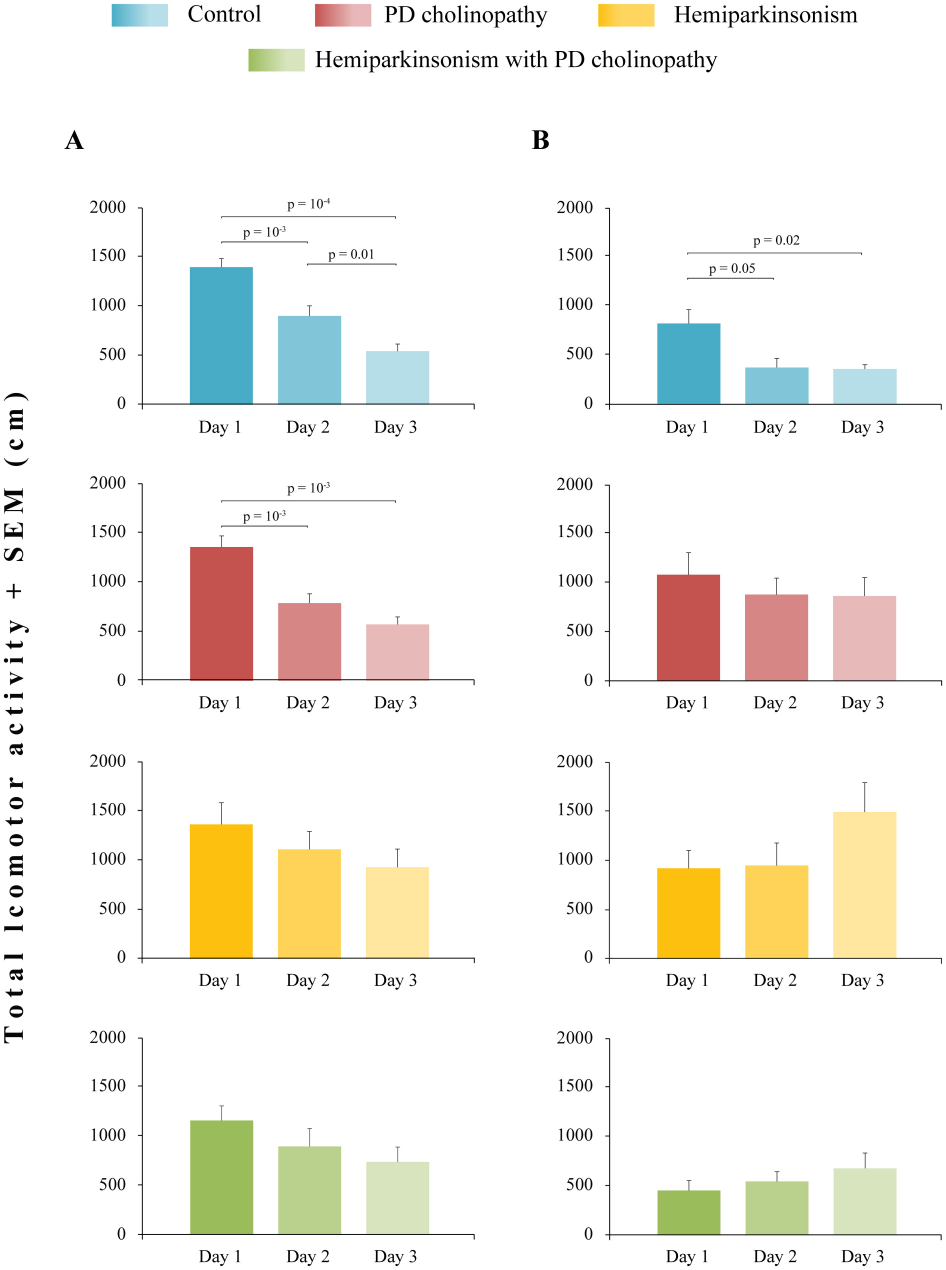
Spatial memory impairment in different rat models of PD neuropathology. **(A)** Spatial habituation test (SHT) 14 days after lesions in control (*n* = 14), PD cholinopathy (*n* = 10), hemiparkinsonism (*n* = 7), and hemiparkinsonism with PD cholinopathy (*n* = 6). There was difference in total locomotor activity in control group (χ^2^ = 24.41; *p* = 10^−4^) and PD cholinopathy (χ2 = 15.14; *p* = 10^−3^); **(B)** Spatial habituation test (SHT) 42 days after lesions in control (*n* = 6), PD cholinopathy (*n* = 5), hemiparkinsonism (*n* = 5), and hemiparkinsonism with PD cholinopathy (*n* = 5). There was difference in total locomotor activity in control group (χ^2^ = 6.82; *p* = 0.03). All individual *p*-values for the Mann-Whitney U tests are shown. All parkinsonian rats showed spatial memory impairment, specifically expressed as a long-lasting memory deficit in hemiparkinsonian rats and as a delayed memory deficit in PD cholinopathy.

In this study, SHT was used as an indirect measure of spatial memory abilities, with habituation defined as a decrease in total distance traveled across the three testing sessions in the open field. As expected, a decrease in locomotor activity during SHT was evidenced in control rats, at both day 14 and day 42, indicating physiological habitual response (Figures 5A, B. χ^2^ ≥ 6.82; *p* ≤ 0.03; z ≥ −4.41; *p* ≤ 0.05). PD cholinopathy also showed physiological habituation, but only 14 days after the lesion (Figure 5A, χ^2^ = 15.14; *p* = 10^−3^; z ≥ −3.48; *p* ≤ 0.01), while 4 weeks later there was no decrease in locomotor activity during SHT (Figure 5B, χ^2^ = 0.38; *p* = 0.83). This delayed lack of inter-session habituation in PD cholinopathy indicated spatial memory impairment. However, both groups of hemiparkinsonian rats (hemiparkinsonism and hemiparkinsonism with cholinopathy) showed no change in locomotor activity during SHT at 14 and 42 days after lesions (Figure 5, χ^2^ ≥ 2.87; *p* ≥ 0.22 for hemiparkinsonism; χ^2^ ≥ 0.72; *p* ≥ 0.22 for hemiparkinsonism with PD cholinopathy), indicating long-lasting spatial memory impairment.

### 3.4 Recognition memory impairment in different rat models of PD

Next, we tested short- and long-term hippocampus-dependent recognition memory in experimental models of PD. Since our modified NORT protocol included long, fixed session duration, we first analyzed the overall exploration dynamics during the 20-min sessions in each experimental group.

**Figure 6:**
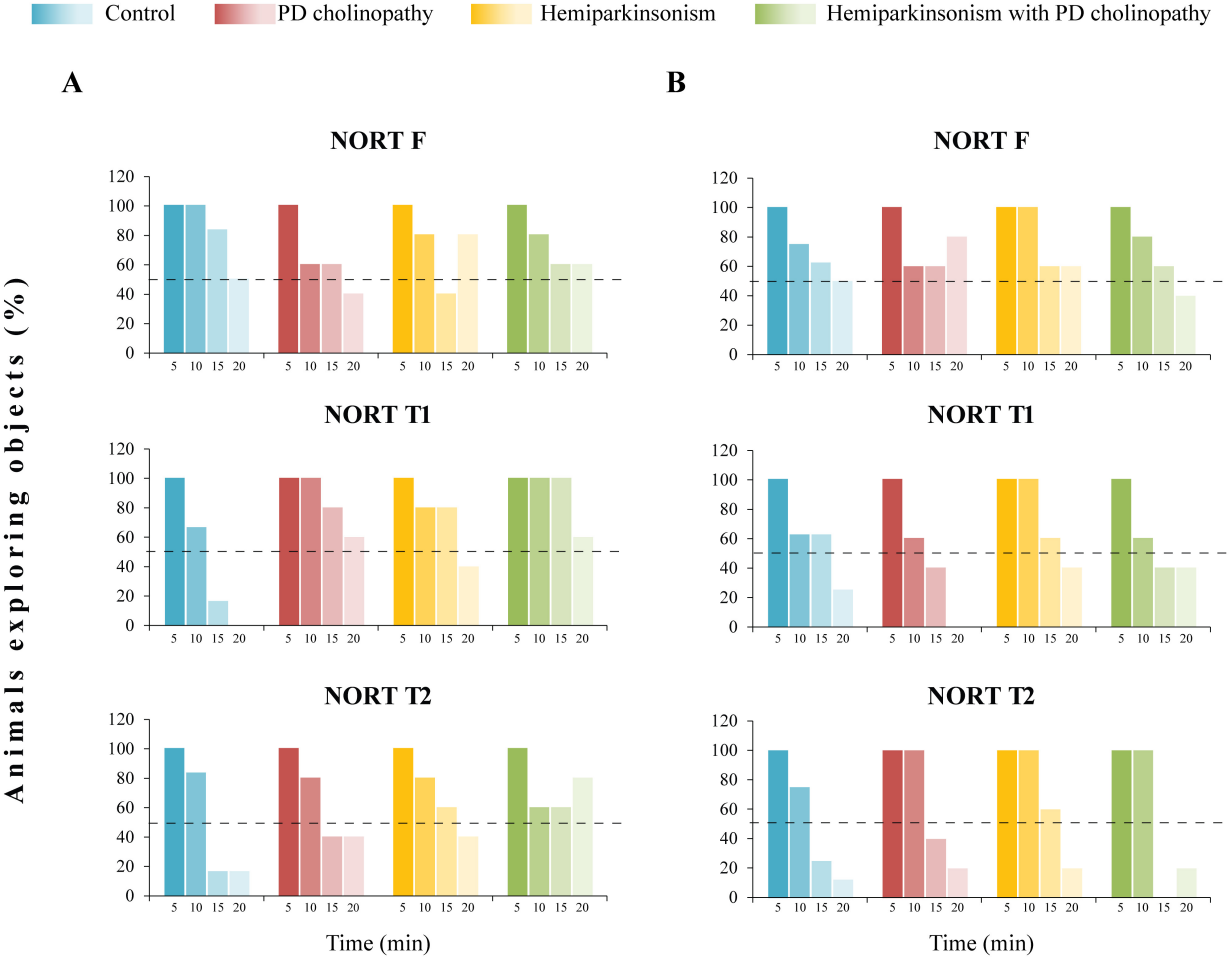
General explorative activity during NORT in different rat models of PD neuropathology. **(A)** Group explorative activity during the 5-min intervals of NORT in control (*n* = 6), PD cholinopathy (*n* = 5), hemiparkinsonism (*n* = 5), and hemiparkinsonism with PD cholinopathy (*n* = 5) 14 days after lesion; **(B)** Group explorative activity during the 5-min intervals of NORT in control (*n* = 8), PD cholinopathy (*n* = 5), hemiparkinsonism (*n* = 6), and hemiparkinsonism with PD cholinopathy (*n* = 5) 42 days after lesion. Data are presented as the percentage of n within the corresponding experimental group, shown with column bars. The dashed line indicates the threshold level set at 50%. A group was considered active when its activity was above the threshold during a given 5-min interval. A group was considered non-active when its activity was below the threshold during a given 5-min interval. NORT F, familiarization phase; NORT T1, test phase 1; NORT T2, test phase 2. The most intensive exploration, with all animals active, occurred during the first 5-min interval, regardless of experimental group or test phase. At day 14, all parkinsonian rats showed prolonged exploration during NORT T1, but at day 42 prolonged exploration was observed only in hemiparkinsonian rats.

**Figure 7:**
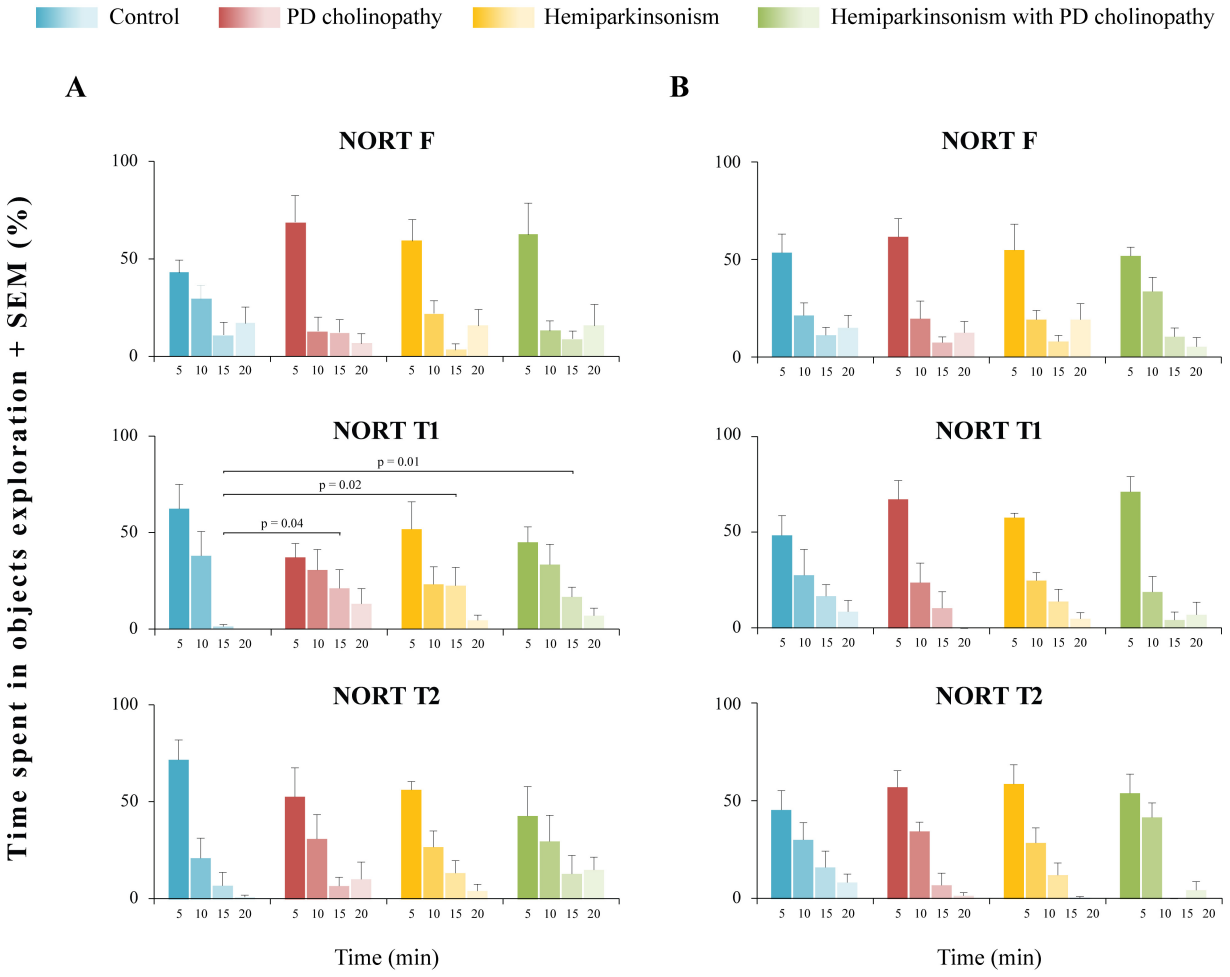
Timeline of exploratory activity during NORT in different rat models of PD neuropathology. **(A)** Group timeline of exploration during the 5-min intervals of NORT in control (*n* = 6), PD cholinopathy (*n* = 5), hemiparkinsonism (*n* = 5), and hemiparkinsonism with PD cholinopathy (*n* = 5) 14 days after lesions. There was difference in exploratory activity only during 15-min time interval in NORT T1 (χ^2^ = 8.16, *p* = 0.04); **(B)** Group timeline of exploration during the 5-min intervals of NORT in control (*n* = 8), PD cholinopathy (*n* = 5), hemiparkinsonism (*n* = 6), and hemiparkinsonism with PD cholinopathy (*n* = 5) 42 days after lesions. There was no difference in exploratory activity between the groups in any 5-min interval (χ^2^ ≥ 0.42, *p* ≥ 0.18). All individual *p*-values for the Mann-Whitney U tests are depicted. NORT F, familiarization phase; NORT T1, test phase 1; NORT T2, test phase 2. The most intensive exploration, accounting for approximately 50% of total exploratory time, occurred during the first 5-min interval, regardless of experimental group or test phase. At day 14, all parkinsonian rats showed prolonged exploration during NORT T1, but at day 42, there was no difference in exploration time between the groups.

Based on general exploration activity during NORT, we identified the first 5-min interval as period of the most intensive exploration, regardless of experimental group or test phase (Figures 6, 7). During that period, all animals were active (group activity 100%), spending about 50% of their total exploratory time. After the first 5-min interval, exploratory activity progressively decreased in all experimental groups, though some specific features were observed.

Two weeks after lesions, all experimental groups were active (>50% group activity) for 15 min during familiarization (Figure 6A, NORT F), with the most intensive exploration in the first 5 min (Figure 7A, NORT F). However, there was no statistically significant difference in exploration time between the groups during each 5-min interval (χ^2^ ≥ 1.09, *p* ≥ 0.36). During Test 1, control rats were actively exploring for only 10 min, whereas all parkinsonian rats had prolonged exploration lasting the full 20 min (Figure 6A, NORT T1**)** The difference in exploration time was evidenced during the 15-min interval, when all parkinsonian rats spent more time in object exploration in contrast to controls (Figure 7A, NORT T1, χ^2^ = 8.16, *p* = 0.04; z ≥ −2.68, *p* ≤ 0.04). During Test 2, both control and PD cholinopathy groups showed intensive exploratory activity for 10 min, while hemiparkinsonian rats again exhibited prolonged exploration over 20 min (Figure 6A, NORT T2**)** However, there was no statistically significant difference in exploration time between the groups during each 5-min interval (Figure 7A, NORT T2; χ^2^ ≥ 0.35, *p* ≥ 0.16).

Four weeks after lesions, all experimental groups were active (> 50% group activity) for a total of 20 min during familiarization (Figure 6B, NORT F), with the most intensive exploration occurring in the first 5 min (Figure 7B, NORT F). However, there was no statistically significant difference in exploration time between the groups during each 5-min interval (χ^2^ ≥ 0.46, *p* ≥ 0.51). During Test 1, control and hemiparkinsonian rats actively explored for 15 min, while PD cholinopathy and hemiparkinsonism with PD cholinopathy groups explored for only 10 min (Figure 6B, NORT T1**)** although difference in exploration time between the groups was not statistically significant (Figure 7B, NORT T1; χ2 ≥ 0.54, *p* ≥ 0.42). During Test 2, all experimental groups actively explored for 10 min (Figure 6B, NORT T2) with no difference in exploration time (Figure 7B, NORT T2; χ2 ≥ 1.24, *p* ≥ 0.18).

**Figure 8:**
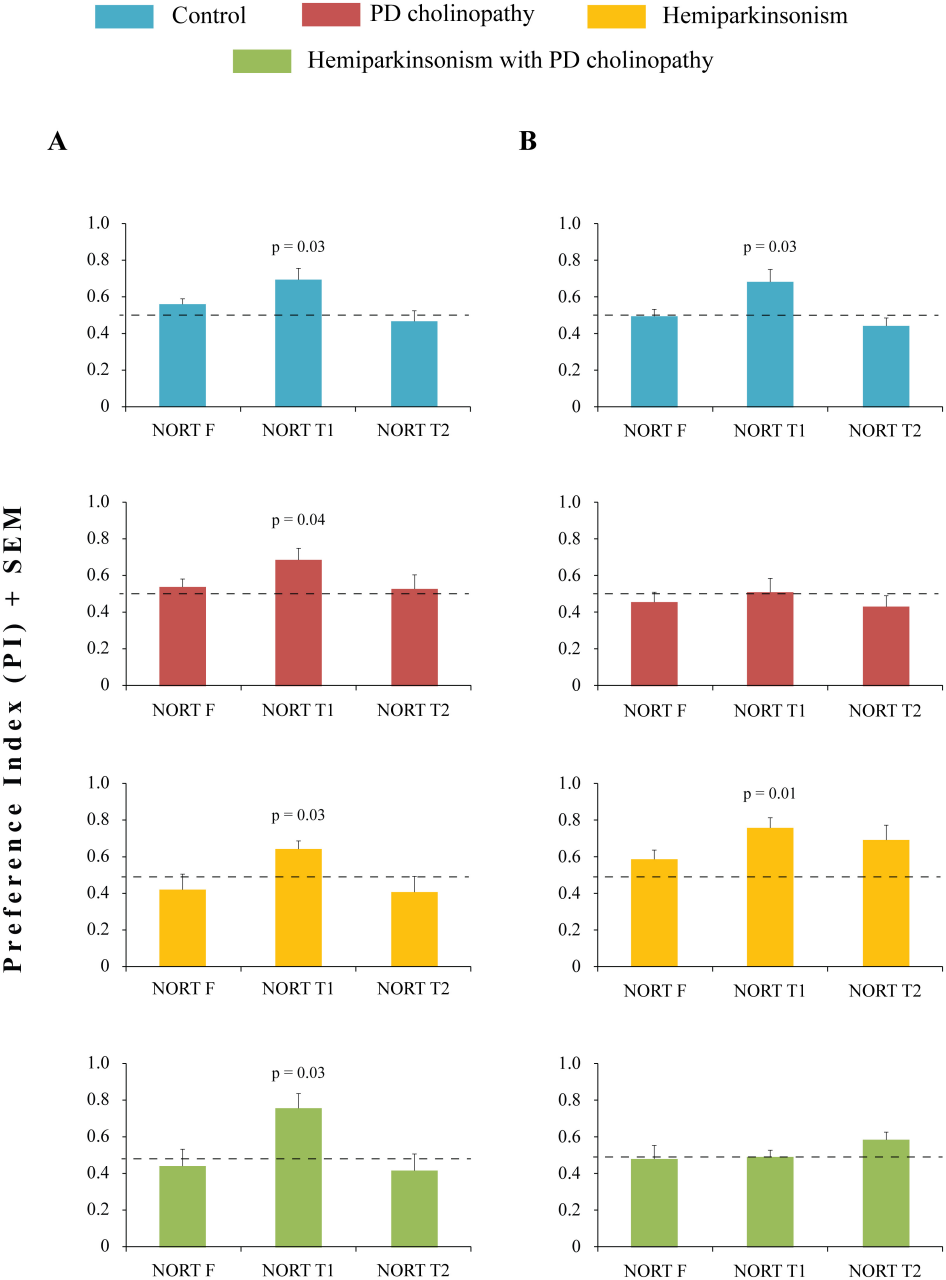
Recognition memory impairment in different rat models of PD neuropathology. **(A)** Preference index (PI) during NORT in control (*n* = 6), PD cholinopathy (*n* = 5), hemiparkinsonism (*n* = 5), and hemiparkinsonism with PD cholinopathy (*n* = 5) 14 days after lesions. During NORT T1, PIs were above chance in all experimental groups (t ≥ 2.96, *p* ≤ 0.04); **(B)** Preference index (PI) during NORT in control (*n* = 8), PD cholinopathy (*n* = 5), hemiparkinsonism (*n* = 6), and hemiparkinsonism with PD cholinopathy (*n* = 5) 42 days after lesions. During NORT T1, PIs were above chance in control (t = 2.66, *p* = 0.03) and hemiparkinsonism (t = 4.62, *p* = 0.01). NORT F, familiarization phase; NORT T1, test phase 1; NORT T2, test phase 2. PIs were compared to chance levels (PI = 0.5) using one-sample *t*-test. Normality of NORT data set was assessed by the Shapiro-Wilk test (W ≥ 0.79, *p* ≥ 0.07 for A; W ≥ 0.69, *p* ≥ 0.12 for B). All individual *p*-values for one sample *t*-test are shown. At day 14, all parkinsonian rats showed intact recognition memory. However, 4 weeks later (day 42), we evidenced recognition memory impairment in PD cholinopathy and hemiparkinsonism with PD cholinopathy.

Based on these results, PI was calculated for the first 5-min interval for all test phases, including familiarization (to assess place preference) (Figure 8). All animals showing no preference for either object during NORT F (PI ≈ 0.5; Figure 8 NORT F; t ≥ −0.97, *p* ≥ 0.11) were included in further short-term (NORT T1) and long-term (NORT T2) recognition memory analysis.

We found no deficit in recognition memory 14 days after lesions (Figure 8A). All experimental groups showed a preference for the novel object during NORT T1 (Figure 8A, NORT T1; t ≥ 2.96, *p* ≤ 0.04), indicating intact short-term recognition memory, and no preference for objects during NORT T2 (Figure 8A, NORT T2; t ≥ −1.11, *p* ≥ 0.33), indicating intact long-term recognition memory.

However, 42 days after lesions, PD cholinopathy and hemiparkinsonism with PD cholinopathy experimental groups showed a recognition memory deficit, with no preference for objects during either NORT T1 (Figure 8B NORT T1; t ≥ −0.24, *p* ≥ 0.82) or NORT T2 (Figure 8B NORT T2; t ≥ −1.16, *p* ≥ 0.12). In contrast, controls and hemiparkinsonism groups showed a preference for the novel object during NORT T1 (Figure 8B, NORT T1; t ≥ 2.66, *p* ≤ 0.03) and no preference for objects during NORT T2 (Figure 8B NORT T2; t ≥ −1.37, *p* ≥ 0.08), indicating intact short- and long-term recognition memory.

### 3.5 Alteration of hippocampal PV+ neurons in different rat models of PD

**Figure 9:**
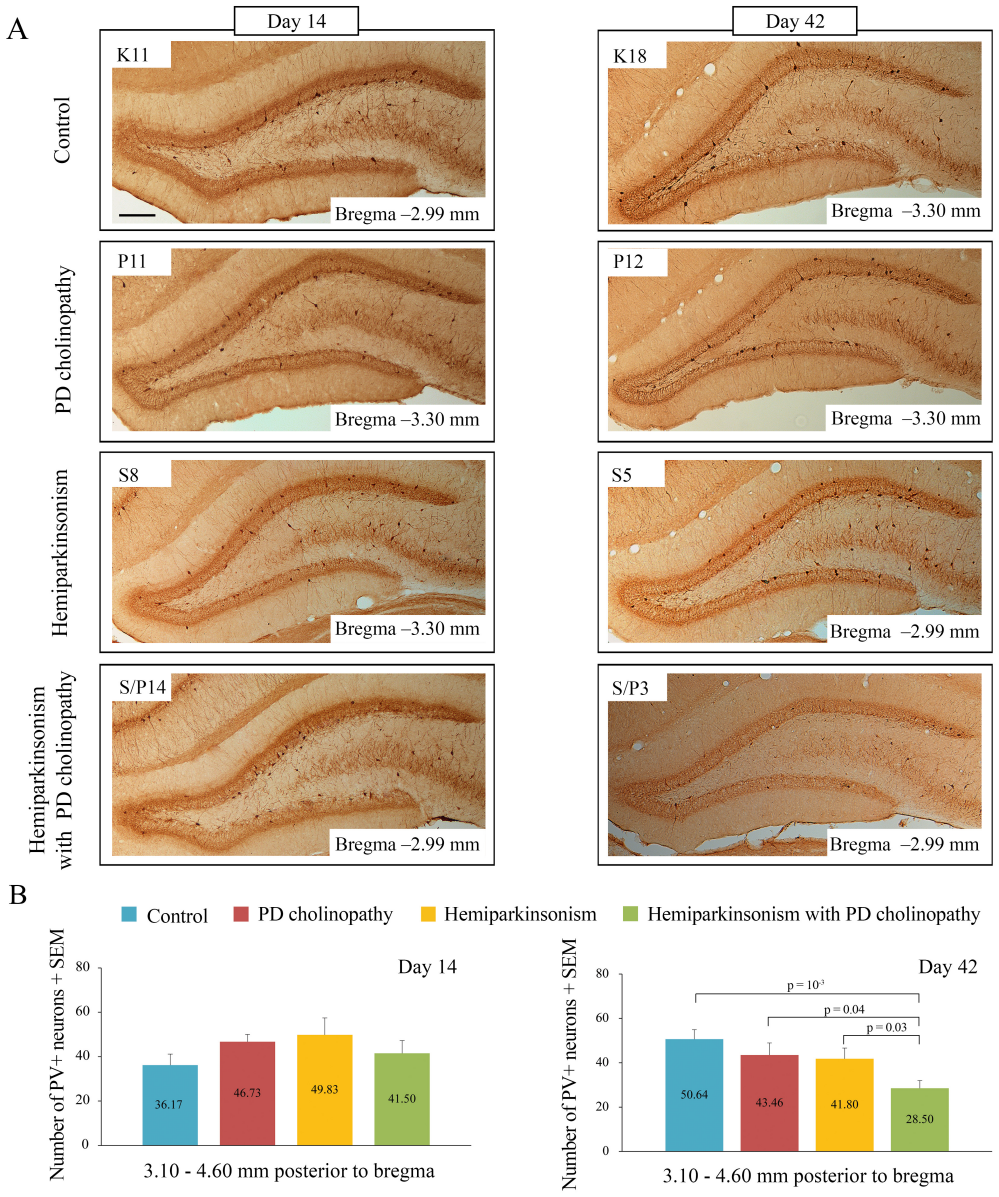
Alteration of hippocampal PV+ neurons in different rat models of PD neuropathology. **(A)** Representative examples of hippocampal PV immunohistochemistry in control (K11, K18), PD cholinopathy (P11, P12), hemiparkinsonism (S8, S5), and hemiparkinsonism with PD cholinopathy (S/P14, S/P3) at 14 and 42 days after lesions. Scale bar: 200 μm; **(B)** Group mean number of hippocampal PV+ neurons within the DG at 14 and 42 days after lesions in control (n_d14_ = 4; n_d42_ = 7), PD cholinopathy (n_d14_ = 6; n_d42_ = 7), hemiparkinsonism (n_d14_ = 3; n_d42_ = 5), and hemiparkinsonism with PD cholinopathy (n_d14_ = 5; n_d42_ = 7). There was no difference in the number of PV+ neurons between groups at day 14 (χ^2^ = 3.13, *p* = 0.37), but there was a difference between groups at day 42 (*p* = 0.01). All individual *p*-values for the Mann-Whitney U test are shown. There was a delayed suppression of PV+ neurons within the DG of the hippocampus in hemiparkinsonism with PD cholinopathy 42 days post-lesion.

To investigate the underlying cellular neuropathology of hippocampus-dependent memory impairment in distinct rat models of PD, we examined alteration in hippocampal PV+ neurons. Representative examples of hippocampal PV immunohistochemistry for all experimental groups at 14 and 42 days post-lesion, along with PV+ quantification are shown in Figure 9. While PD cholinopathy and hemiparkinsonism experimental groups showed no alteration in PV+ neurons at 14 or 42 days post-lesion (Figure 9, z ≥ −1.91, *p* ≥ 0.06), hemiparkinsonism with PD cholinopathy group exhibited delayed suppression of PV+ neurons in the DG (Figure 9; z = −3,17, *p* = 10^−3^) at 42 days post-lesions.

**Figure 10:**
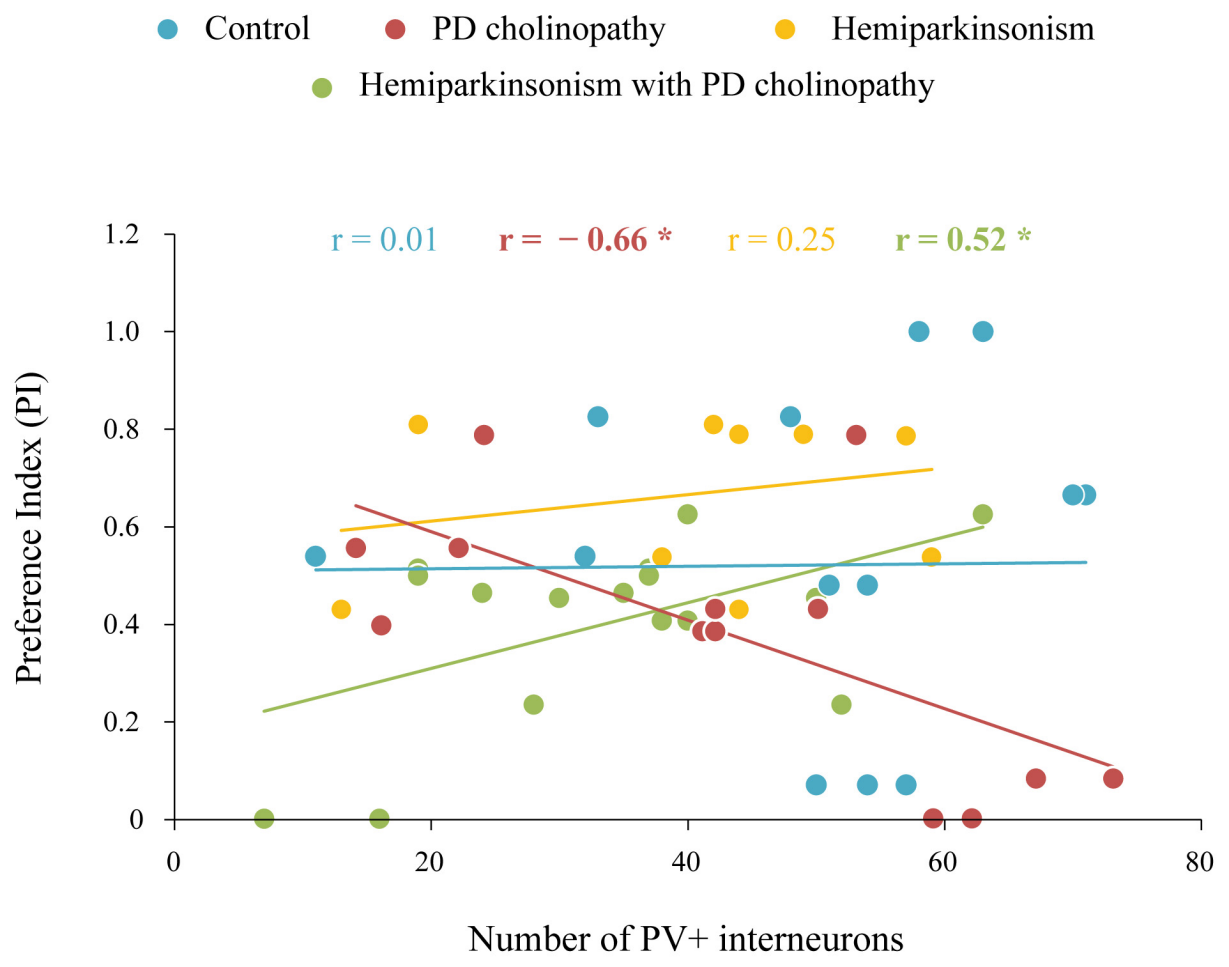
Functional coupling between hippocampal PV+ neurons and recognition memory deficit in distinct rat models of PD neuropathology. Correlation analysis between the number of hippocampal PV+ neurons in the DG and the preference index (PI) during NORT T1 at day 42 in control (*n* = 8), PD cholinopathy (*n* = 7), hemiparkinsonism (*n* = 7), and hemiparkinsonism with cholinopathy (*n* = 8). Bold numbers with asterisks indicate statistically significant correlations at *p* ≤ 0.05. Short-term recognition memory impairment in hemiparkinsonism with PD cholinopathy was positively correlated with a decreased number of hippocampal PV+ neurons 42 days post-lesion.

To further investigate the underlying PV+ neuron alteration associated with hippocampus-dependent memory impairments in distinct rat models of PD, we correlated the number of PV+ neurons and PIs from short-term (NORT T1) and long-term (NORT T2) memory tests 42 days after lesions. This correlation analysis revealed a significant positive correlation between PV+ neuron suppression and short-term recognition memory impairment in hemiparkinsonism with PD cholinopathy (Figure 10, *r* = 0.52, *p* = 0.04). We also found a significant negative correlation between the number of PV+ neurons and short-term memory impairment in PD cholinopathy (Figure 10 *r* = −0.66, *p* = 0.01). Conversely, there was no functional coupling between the number of PV+ neurons and long-term memory impairment in our experimental models of PD 42 days after lesions (*r* ≥ −0.56, *p* ≥ 0.05; data not shown).

## 4 Discussion

In this study, we investigated spatial and recognition hippocampus-dependent memory deficits along with changes in hippocampal PV+ neurons in distinct experimental models of PD. Our PD models showed no motor deficits (Figure 4) but exhibited spatial (Figure 5) and recognition memory impairments (Figure 8), which were associated with suppression of hippocampal PV+ neurons only in hemiparkinsonism with PD cholinopathy (Figures 9, 10).

Specifically, although hippocampal PV expression remained unchanged over time in PD cholinopathy, we observed delayed impairments in spatial and both short- and long-term recognition memory. In hemiparkinsonian rats, unchanged hippocampal PV expression was followed by persistent spatial memory impairment, while recognition memory remained intact over time. In hemiparkinsonian rats with PD cholinopathy, persistent spatial memory impairment was followed by delayed short- and long-term recognition memory deficits, along with hippocampal PV suppression, which was functionally linked to recognition memory impairment (Figure 10).

PD is primarily characterized by motor system impairment. Decreased motor activity, especially bradykinesia and rigidity, is one of the core symptoms of PD and is predominantly a consequence of severe loss of dopaminergic innervation (Błaszczyk, 2016; Schapira et al., 2017). In addition, postural instability and gait disorder in PD have been strongly correlated with cholinergic dysfunction (Bohnen et al., 2022; Perez-Lloret and Barrantes, 2016). In particular, thalamic cholinergic denervation induced by PPT cholinergic cell loss has been associated with gait and balance problems in PD, including frequent falls (Müller and Bohnen, 2013). Since our experimental PD models were based on dopaminergic and/or cholinergic lesions, we assessed basal locomotor activity to determine any lesion-induced differences in motor performance. This is also important for other behavioral assessments that rely on initial exploratory activity levels (SHT and NORT). However, we did not evidence any motor deficit. Partial DA and/or Ach depletion with > 50% neuronal loss in the SNpc and > 27% neuronal loss in the PPT, as evident in our experimental models (Figure 3), did not affect spontaneous locomotor activity 14 and 42 days after lesions, as all experimental groups demonstrated the same level of motor performance (Figure 4). This absence of a motor deficit in our study is preferred rather than unexpected. The extent of lesions in neurotoxin-based models of PD varies in a dose-dependent manner, with substantial lesions resulting in severe motor deficit, and representing an advanced (late) stage of the disease (Duty and Jenner, 2011). To mimic the earlier (premotor) stage of PD, we deliberately aimed for partial lesions to study non-motor features of PD without possible interference from motor symptoms.

Habituation to a spatial stimulus is one of the simplest forms of non-associative hippocampus-dependent learning in rodents (Leussis and Bolivar, 2006). Based on the natural preference for novelty, spatial habituation measures changes in exploratory behavior in response to prolonged or repeated exposure to a novel environment. Generally, when repeatedly placed in the same environment, rodents display a decrease in activity over time (usually measured as distance traveled during the OFT) both within a single session (intra-session habituation) and across sessions (inter-session habituation). It has been suggested that intra-session habituation measures adaptivity to the environment (learning), while inter-session habituation measures long-term memory of previous exposure (Bolivar, 2009). Therefore, in this study, SHT was used to assess the animals' ability to learn and remember spatial information (empty OF arena) and served as an indirect measure of spatial memory abilities. Our findings indicate that all experimental groups demonstrated an identical pattern of intra-session habituation (decrease in locomotor activity over 30 min of OFT) at both 14 and 42 days following lesions (Figure 4, left panels). However, inter-session habituation was impaired. The lack of habituation in hemiparkinsonian rats (hemiparkinsonism and hemiparkinsonism with PD cholinopaty) at 14 and 42 days following lesions suggests long-lasting spatial memory impairment (Figure 5). In contrast, lack of habituation in PD cholinopathy was evidenced only 42 days following lesion, indicating delayed spatial memory impairment (Figure 5).

Spatial memory, defined as the ability to encode, store, and retrieve information about environmental spatial orientation, is a critical component of daily functioning and has been found to be impaired in PD (García-Navarra et al., 2025). Current findings suggest that spatial memory deficits in PD result from disruptions in multiple neural circuits, including the hippocampus, basal ganglia, dopaminergic pathways, and frontal cortical regions (García-Navarra et al., 2025). The hippocampus plays a crucial role in spatial memory, while the basal ganglia, particularly the striatum, are implicated in procedural learning and spatial navigation. Interactions between the hippocampus and basal ganglia are mediated by DA. In PD, progressive loss of DA neurons within the SNpc and consequent striatal DA depletion compromise the integrity of these interactions, contributing to deficits in spatial memory (García-Navarra et al., 2025). The long-lasting impairment of spatial memory in hemiparkinsonian rats demonstrated in this study supports these findings and suggests an important role of dopaminergic denervation in spatial memory deficits in the prodromal stage of PD.

The most common cognitive symptom in early-diagnosed PD patients is episodic (recognition) memory impairment (Das et al., 2019; Muslimovic et al., 2005). Recognition memory is necessary to distinguish novel information from what is already known. It has been proposed that memory for objects in rodents is similar to episodic memory in humans (Dere et al., 2005). Spontaneous recognition tests, such as the NORT, have been used to evaluate non-associative recognition memory in rodents for decades (Ennaceur, 2010; Ennaceur and Delacour, 1988). Following the familiarization phase, the behavior of animals during NORT T1 reflected recognition and exploration of novelty (new learning during retrieval) and during NORT T2 the memory of previously experienced novelty.

It has been demonstrated that partial DA depletion induced by bilateral striatal 6-OHDA lesions in mice leads to impairment of long-term recognition memory (Bonito-Oliva et al., 2014). However, in our study, recognition memory was preserved in all experimental groups at the beginning of follow-up period (Figure 8A). This indicates that the novel object was assessed, explored, and became familiar during NORT T1 (short-term memory intact), and was therefore remembered and treated as familiar during NORT T2 (long-term memory intact). At the same time, overall exploration dynamics analysis revealed prolonged exploration during NORT T1 in all PD models compared to controls (Figures 6A, 7A). The amount of time spent exploring an object may be directly proportional to the strength and detail of the memory formed (Cohen and Stackman, 2015). Therefore, it is possible that prolonged exploration during the 20-min NORT T1 period in parkinsonian rats (compared to the 10-min period in the control group) contributed to memory strength and compensated for a possible memory deficit. If our NORT protocol had included shorter session durations, we might have detected this memory deficit, but in that case, the compensatory effect of prolonged exploration would have gone unnoticed. However, 42 days after lesions, rats with PD cholinopathy and hemiparkinsonism with PD cholinopathy displayed both short- and long-term recognition memory deficits (Figure 8B), indicating failure of memory retrieval of previously explored objects. At this time, prolonged exploratory activity was observed only in hemiparkinsonian rats, which demonstrated intact recognition memory (Figures 6B, 7B). These results suggest that a selective reduction of cholinergic, but not dopaminergic, innervation was sufficient to induce delayed recognition memory impairment.

Behavioral assessments in this study are based on initial exploratory activity levels, which may vary among experimental models of PD due to lesion-induced motor deficits. Low locomotor activity can affect both habituation and object exploration during the familiarization phase. Consequently, a lack of habituation or object recognition may not result from memory deficits per se, but rather from incomplete initial exploration of the open field area or objects. However, as previously stated, we did not evidence any baseline differences in motor performance. This excludes the possibility that the deficits in spatial, short- and long-term recognition memory observed in our experimental models of PD are caused by reduced exploratory activity.

The hippocampus plays a key role in memory formation, and both spatial and recognition memory are dependent on the hippocampus (Cohen and Stackman, 2015; Leussis and Bolivar, 2006). Multiple functional and structural imaging studies have linked memory impairment and progression to dementia with hippocampal hypometabolism and atrophy in PD patients (González-Redondo et al., 2014; Martín-Bastida et al., 2021). Moreover, the degree of hippocampal atrophy is strongly correlated with the degree of memory impairment (Kandiah et al., 2014). Although few in number, hippocampal PV+ neurons are critical for memory formation and information processing (Ognjanovski et al., 2017; Verret et al., 2012; Yi et al., 2014). Specifically, the consolidation and retrieval of memories depend on neuronal circuits in the hippocampus, whose neuronal communication, E/I balance and GABAergic disinhibition are regulated by PV+ neurons (Nahar et al., 2021; Sohal et al., 2009). In addition, PV+ neurons generate gamma oscillations, which are essential for normal brain function and cognition, especially for cortical information processing (Bartos et al., 2007; Cardin et al., 2009; Gulyás et al., 2010; Nahar et al., 2021). A recent study suggests that immediately following learning, hippocampal PV+ neurons drive local oscillations and the reactivation of local neuronal populations, directly promoting network plasticity and long-term memory formation (Ognjanovski et al., 2017). Deficits in inhibitory activity due to alterations in PV+ neurons lead to network dysfunction. Thus, impairment of GABAergic inhibition and the resulting E/I imbalance cause hyperexcitability and desynchronization of neuronal networks, leading to impaired information processing, learning, and memory formation (Hollnagel et al., 2019). In our study, only hemiparkinsonism with PD cholinopathy induced a delayed suppression of PV+ neurons within the DG (Figure 9), which was functionally coupled with delayed recognition memory impairment (Figure 10). These results indicate that this delayed, PV+ neuron-mediated alteration of an inhibitory transmission in the hippocampus is associated with memory impairment and suggest that synergistic dysfunction across multiple neurotransmitter systems, particularly the dopaminergic and cholinergic interplay observed in our study, underlies memory deficit in the progression of this PD neuropathology.

Hippocampal GABAergic interneurons are diverse and differ in their functional properties, axonal arborization, and expression of molecular markers (Bartos et al., 2007). In this research, we focused on PV neurons, which are particularly important for recognition memory tasks that depend on fast, temporally precise hippocampal signaling (Royer et al., 2012). These fast-spiking interneurons form perisomatic synapses, primarily targeting the soma and axon initial segment of pyramidal cells, and are essential for generating oscillatory activity in the gamma-frequency range (Bartos et al., 2007; Donato et al., 2013). Gamma oscillations coordinate the precise timing of neuronal activity during memory processes such as encoding and retrieval (Bartos et al., 2007; Ognjanovski et al., 2017). Therefore, alterations in PV+ interneurons are likely to directly affect the delayed recognition memory deficits observed in hemiparkinsonism with PD cholinopathy, highlighting their functional specificity. Other interneuron subtypes, such as non-fast-spiking somatostatin-expressing (SST+) cells, primarily target the distal dendrites of pyramidal cells and generate beta-frequency oscillations, modulating synaptic integration rather than timing precision (Royer et al., 2012). These interneurons are thought to contribute to the formation of contextual fear memory (Lovett-Barron et al., 2014) and object location learning (Honoré and Lacaille, 2022). The precise contribution of other subtypes of hippocampal GABAergic interneurons to memory impairment in our experimental PD models remains to be determined.

The association between the number of PV+ neurons and recognition memory performance was evidenced following lesion-induced pathology, primarily related to cholinergic denervation (Figure 10). Unchanged PV+ expression was not associated with recognition memory performance in hemiparkinsonism, indicating that a dopaminergic deficit greater than 50% is not sufficient to induce this functional coupling. In contrast, the association between PV+ expression and recognition memory in PD cholinopathy and hemiparkinsonism with PD cholinopathy appeared following a cholinergic deficit greater than 27% (Figure 3A). Although cholinergic denervation was required to induce recognition memory impairment, it was not sufficient to reduce PV+ expression. Thus, unchanged PV+ expression was negatively correlated with recognition memory impairment in PD cholinopathy, indicating better recognition memory performance in rats with fewer PV+ neurons. Additional dopaminergic denervation in hemiparkinsonism with PD cholinopathy suppressed PV+ neurons, and in these rats, the degree of PV+ suppression was strongly correlated with the degree of short-term recognition memory impairment. However, it remains unclear whether the PV+ suppression observed in our study reflects a reduction in the number of PV+ cells or changes in their function, and whether an unchanged number indicates unchanged activity. In this context, the negative correlation in PD cholinopathy could result from altered (increased) activity. Therefore, when PV+ neurons are unchanged, impaired recognition memory could benefit from a reduced number of hyperactive PV+ neurons. Conversely, when PV+ neurons are suppressed, the remaining (possibly hyperactive) PV+ neurons may support recognition memory and compensate for impaired function.

Neurodegenerative diseases are particularly linked to hemispheric asymmetries, with the most significant finding being the role of lesion laterality (Mundorf and Ocklenburg, 2023). In PD, disease severity and symptoms depend on the affected hemisphere. Animal models of PD are often created by inducing a unilateral lesion in the right nigrostriatal pathway, resulting in motor impairments similar to those observed in patients with PD, including asymmetric use of the ipsilateral paw (Mendes-Pinheiro et al., 2021) and asymmetry in temporal and spatial walking patterns (Broom et al., 2019). In our study, bilateral PPT cholinergic neuronal loss was equivalent in both the PD cholinopathy model and in hemiparkinsonism with PD cholinopathy, as was unilateral (right) dopaminergic neuronal loss in hemiparkinsonism compared to hemiparkinsonism with PD cholinopathy (Figure 3A). However, we did not assess the effects of these lesions on lateralization at the electrophysiological or behavioral level, particularly the effects of unilateral SNpc dopaminergic lesions. Therefore, we cannot exclude the possibility that unilateral dopaminergic neuronal loss in hemiparkinsonian models leads to compensatory changes in hemispheric lateralization. Dopamine depletion in one hemisphere may differentially disrupt the function of the basal ganglia and frontal cortex on the ipsilateral side and produce compensatory shifts in activation toward motor and non-motor areas of the contralateral hemisphere (Rios et al., 2019; Mundorf and Ocklenburg, 2023). In the parkinsonian state, the motor cortex and basal ganglia undergo dynamic remodeling of movement representation, such as the loss of the normal contralateral lateralized activity pattern (Rios et al., 2019), which may consequently have a different impact on the function of underlying inhibitory hippocampal PV neurons in our distinct rat models of PD neuropathology, particularly on delayed recognition memory impairment in our combined model of PD neuropathology (hemiparkinsonism with PD cholinopathy). Further investigation of this issue is warranted.

Our results show that different PD neuropathologies underlie different memory impairments in rats. While dopaminergic denervation plays an important role in impairing spatial memory from the prodromal stage of PD, cholinergic denervation impairs recognition memory in a delayed manner. However, only their synergistic dysfunction alters hippocampal PV+ neuron-mediated inhibitory transmission during PD progression, which was correlated with memory impairment. Future therapeutic strategies should focus on ameliorating multisystem synergistic dysfunction to prevent or delay the progression of cognitive deficits in PD.

## Data Availability

The raw data supporting the conclusions of this article will be made available by the authors, without undue reservation.
